# Interaction of Myosin Phosphatase Target Subunit (MYPT1) with Myosin Phosphatase-RhoA Interacting Protein (MRIP): A Role of Glutamic Acids in the Interaction

**DOI:** 10.1371/journal.pone.0139875

**Published:** 2015-10-07

**Authors:** Eunhee Lee, Walter F. Stafford, III

**Affiliations:** Cardiovascular Biology Program and AUC Research Laboratory, Boston Biomedical Research Institute, 64 Grove St, Watertown, MA, 02472, United States of America; Russian Academy of Sciences, Institute for Biological Instrumentation, RUSSIAN FEDERATION

## Abstract

Scaffold proteins bind to and functionally link protein members of signaling pathways. Interaction of the scaffold proteins, myosin phosphatase target subunit (MYPT1) and myosin phosphatase-RhoA interacting protein (MRIP), causes co-localization of myosin phosphatase and RhoA to actomyosin. To examine biophysical properties of interaction of MYPT1 with MRIP, we employed analytical ultracentrifugation and surface plasmon resonance. In regard to MRIP, its residues 724–837 are sufficient for the MYPT1/MRIP interaction. Moreover, MRIP binds to MYPT1 as either a monomer or a dimer. With respect to MYPT1, its leucine repeat region, LR (residues 991–1030) is sufficient to account for the MYPT1/MRIP interaction. Furthermore, point mutations that replace glutamic acids 998–1000 within LR reduced the binding affinity toward MRIP. This suggests that the glutamic acids of MYPT1 play an important role in the interaction.

## Introduction

Scaffold proteins do not have intrinsic catalytic activity and control a wide range of signaling networks. They bind to multiple proteins, promote interactions between components, and facilitate signaling pathways. Scaffold proteins are also targets for regulation of signaling pathways, conferring increased flexibility and selectivity of cellular responses (for reviews, [1–3]).

Myosin phosphatase target subunit (MYPT1; a.k.a. myosin binding subunit, MBS) is a scaffold protein. MYPT1 binds to a catalytic subunit (PP1cδ) at its N-terminus and to a small subunit (M20) at its C-terminus, forming a myosin phosphatase holoenzyme. MYPT1 interacts with various signaling proteins and serves as a direct target for the regulation of myosin phosphatase. The MYPT1 gene is conserved and targeted disruption of the MYPT1 gene in mouse leads to the failure of embryonic development [4], implying its essential role. MYPT1 plays a role in smooth muscle contraction, cell migration, cell adhesion, and the cell cycle [5].

Myosin phosphatase-RhoA interacting protein (MRIP) was identified as a RhoA-binding protein from murine tissue (named p116^Rip^ by the authors) [6] and as a MYPT1-binding protein from human studies [7,8]. MRIP is a scaffold protein. The MRIP gene is conserved in a wide range of species, implying its functional importance. Co-localization of MYPT1 and MRIP causes control of signaling pathways, such as stress fiber formation [8], insulin-induced vasodilation [9], and hypertension [10].

Using immunoprecipitation and immunoblotting, different research groups have demonstrated that the full-length MRIP interacts with the full-length MYPT1 [7,11,12]. It is, however, not clear which residues of MRIP are critical for its binding to MYPT1. With respect to MYPT1, immunoprecipitation and immunoblotting experiments showed that leucines within the C-terminal region of MYPT1 are important for its binding to MRIP.

To shed further light on the interaction of MYPT1 with MRIP, we examined biophysical properties of the interaction. Using analytical ultracentrifugation (AUC) and surface plasmon resonance (SPR), we measured binding between purified MRIP- and MYPT1-derived peptides which represent deletions or substitution mutations of the two proteins. We mapped the minimal binding regions and determined the amino acid residues critical for the interaction. This study provides a biophysical insight into the myosin phosphatase signaling cascade.

## Results

### Design Rationale for Recombinant MRIP Peptides

**Fig 1:**
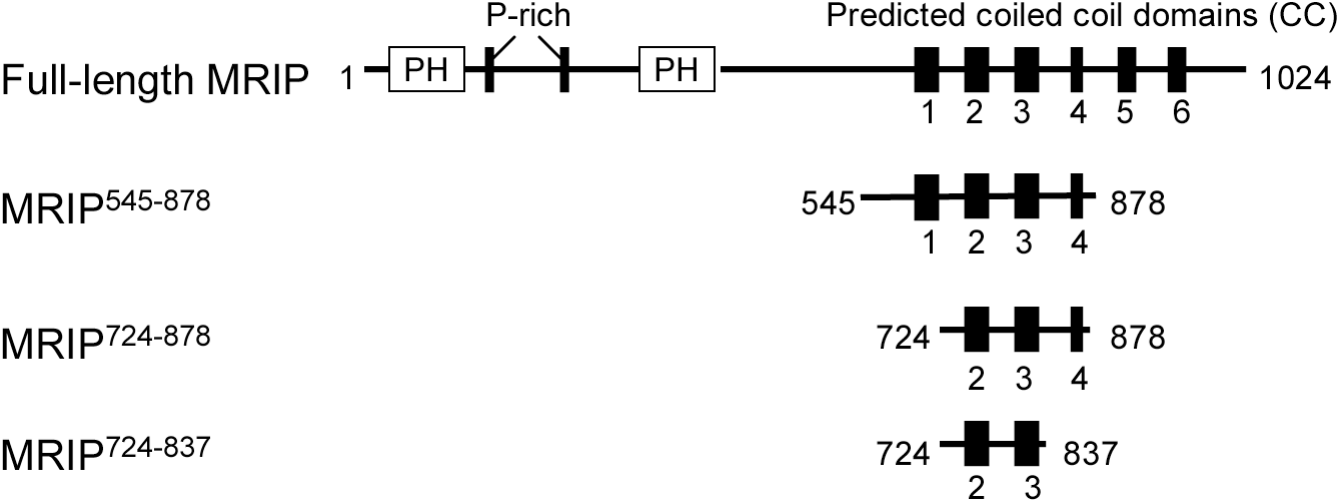
Schematic diagram of MRIP peptides. The peptides were named after the positions of beginning and ending with respect to the full-length position: PH, pleckstrin homology domain (residues 45–148 and 389–480); P-rich, proline-rich domain (residues 164–172 and 284–295); CC1-CC6, coiled coil domains predicted by multicoil program (CC1, resiudes 673–708; CC2, residues 729–769; CC3, residues 791–829; CC4, residues 847–861; CC5, residues 900–927; CC6, residues 946–973).

Using the Intein Mediated Purification with an Affinity Chitin-binding Tag Kit (IMPACT™, New England BioLabs), we purified peptides representing the C-terminus of MYPT1 and a coiled coil region of MRIP. The kit removes the chitin fusion tag to leave a native N-terminus, and thus all recombinant peptides in this study do not have a tag. For the coiled coil region of MRIP, we constructed three MRIP peptides based on the following reason. The analysis of a multicoil program [13] predicted that MRIP has six potential coiled coil domains (CC1, residues 673–708; CC2, residues 729–769; CC3, residues 791–829; CC4, residues 847–861; CC5, residues 900–927; CC6, residues 946–973), and the coiled coil probabilities of domain are 0.98 (CC1), 0.97 (CC2), 0.83 (CC3), 0.68 (CC4), 0.69 (CC5), and 0.55 (CC6) (Fig 1). We hypothesized that the domains CC1-CC4 may contain a critical region involved in both homodimerization and interaction with MYPT1. By virtue of deletions of coiled coil domains, we constructed MRIP^545-878^ (residues 545–878; CC1-CC4), MRIP^724-878^ (residues 724–878; CC2-CC4), and MRIP^724-837^ (residues 724–837; CC2-CC3). All three MRIP peptides were α-helices from circular dichroism spectropolarimetry (data not shown).

### MRIP Forms a Dimer in Solution

**Fig 2:**
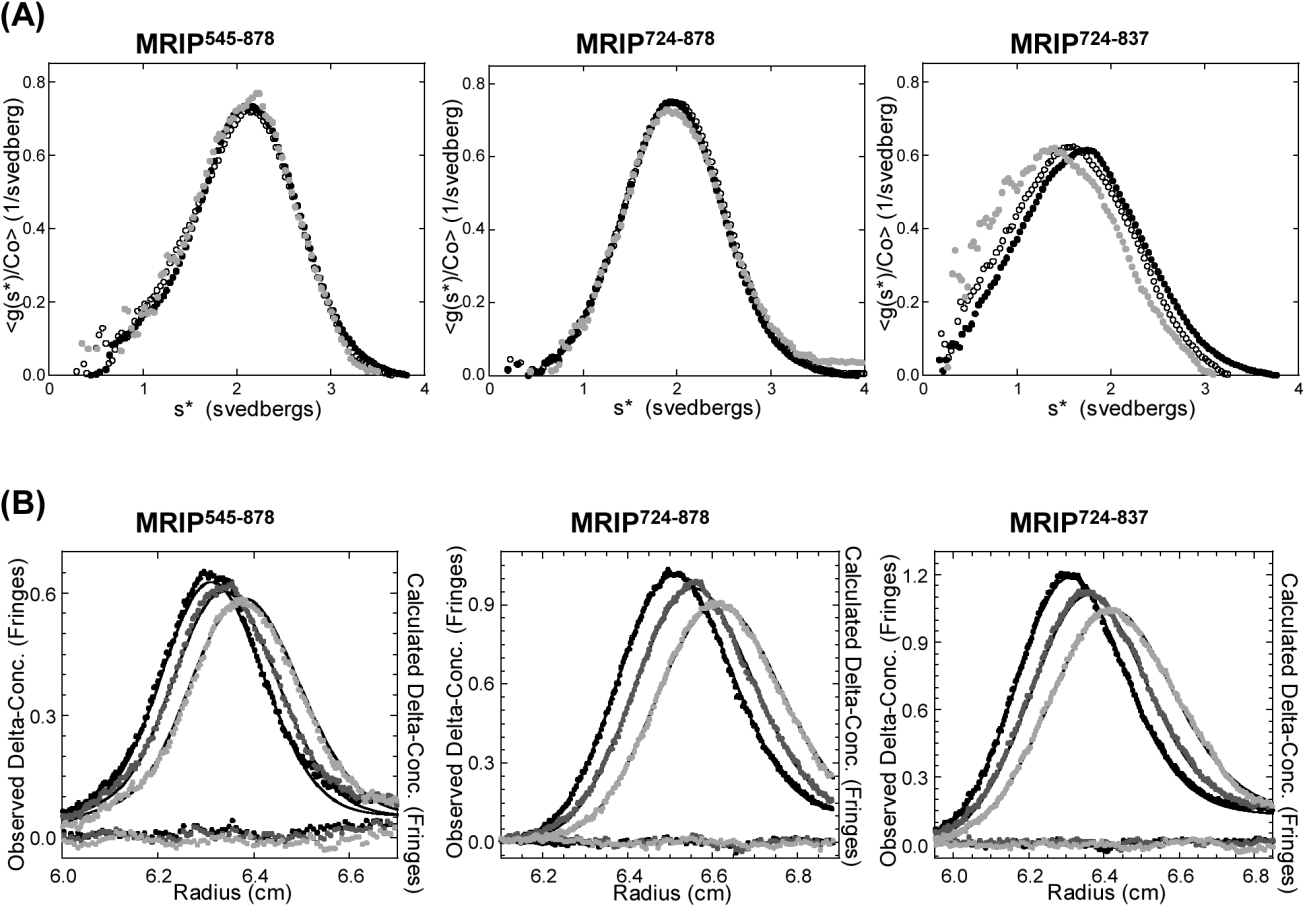
AUC analyses of MRIP peptides. **(A)** The concentration-normalized time derivative g(s*) curves of the peptides: Black filled-in circles, original solutions (MRIP^545-878^, 13.6 μM; MRIP^724-878^, 42.3 μM; MRIP^724-837^, 67.1 μM); open circles, 3-fold dilutions; gray filled-in circles, 9-fold dilutions. **(B)** Curve fitting using SedAnal. Results from global fitting: Closed circles (black, gray, and light gray), observed data; lines (black, gray, and light gray), fits; circles around y = 0, residuals. In most cases the data points cannot be distinguished from the fit.

To examine whether or not the MRIP peptides form dimers in solution, we conducted AUC analyses. Sedimentation velocity was performed over a range of concentrations of MRIP^545-878^ (1.5–13.6 μM), MRIP^724-878^ (4.7–42.3 μM), and MRIP^724-837^ (7.5–67.1 μM) (Fig 2A). The different maximal concentration for each peptide reflects limitation in solubility. The concentration-normalized time derivative g(s*) curves of all concentrations of MRIP^545-878^ or MRIP^724-878^ were super-imposable, indicating no-concentration dependence and the presence of one species in solution. In contrast, g(s*) curves for MRIP^724-837^ showed concentration dependence and a reversible interaction.

To elucidate the stoichiometries of the MRIP peptides, the SedAnal program was used for curve fitting (Fig 2B). Diffusion is explicitly taken into account in the Lamm equation and does not complicate the analysis of the data from sedimentation velocity. Diffusion contains important information about the shape of the molecule and that information is used in the data analysis. The recovery of samples after centrifugation is essentially 99% of the loading materials. In the case of MRIP^545-878^, global fitting to a model of two components yielded a molar mass of 39.4 kDa for one component (confidence level (CL), 0.95; min, 38.1; max, 40.9), close to the formula weight of the monomer (39.0 kDa), and a sedimentation coefficient of 2.17±0.01 S (CL, 0.95). A molar mass of the other component was 21.2 kDa (13% mole/mole), which might have been a contaminant or a product of proteolytic degradation during purification. The root mean square deviation (RMSD) of the fit was 0.010. In short, MRIP^545-878^ forms a monomer in solution. The limited solubility of MRIP^545-878^ prevented us from using a higher concentration and for this reason we cannot exclude the possibility of its forming a dimer. In the case of MRIP^724-878^, global fitting to a single component, single-species ideal model resulted in a molar mass of 38.2±0.3 kDa (CL, 0.95), close to the formula weight of a homodimer (37.4 kDa), and a sedimentation coefficient of 2.007±0.002 S (CL, 0.95). The RMSD of the fit was 0.008. Thus, MRIP^724-878^ forms a tight dimer in solution. In the case of MRIP^724-837^, global fitting to a model of monomer-dimer reaction produced a molar mass of 13.9±0.2 kDa (CL, 0.95), close to the formula weight of a monomer (13.9 kDa). Sedimentation coefficients were 1.29 S (CL, 0.95; min, 1.23; max, 1.31) for the monomer and 1.90 S (CL, 0.95; min, 1.88; max, 1.91) for the dimer. The monomer-dimer dissociation constant K_d_ was 21 μM (CL, 0.95; min, 22; max, 19), and the RMSD of the fit was 0.009.

Three-fold serial dilutions of each peptide covers a somewhat overlapping concentration range but the maximal concentration of MRIP^545-878^ (13.6 μM) was lower than the K_d_ (21 μM) of the monomer-dimer of MRIP^724-837^. Thus, it is possible that 13.6 μM may have been too low to detect dimer, leaving open the question of whether MRIP^545-878^ can dimerize.

### Both Monomeric and Dimeric MRIP Bind to MYPT1

**Fig 3:**
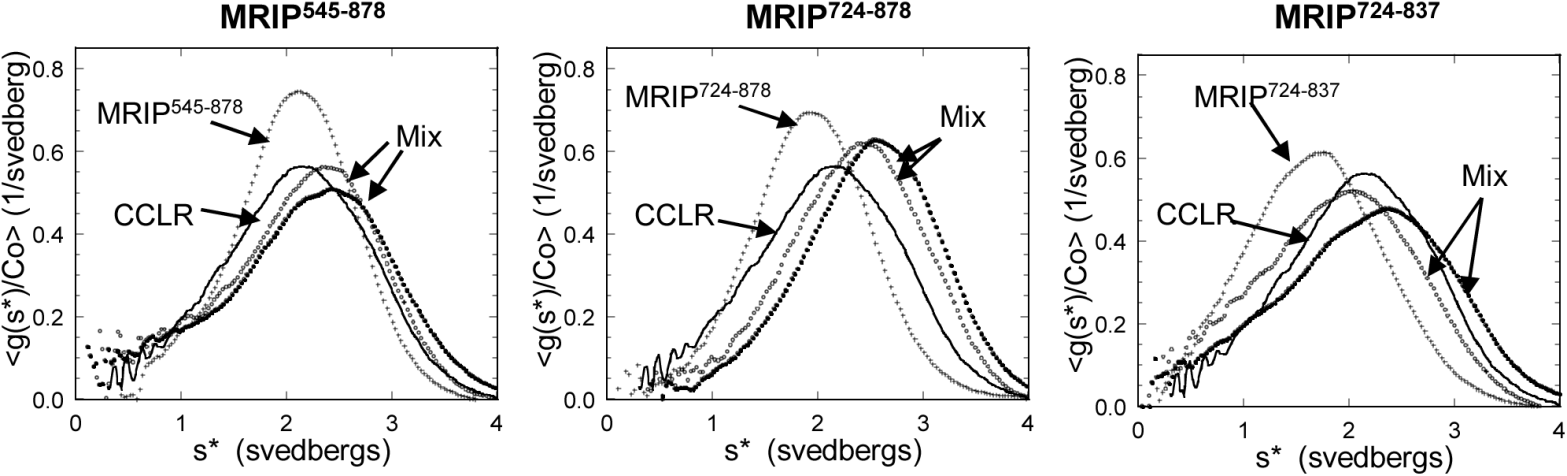
All MRIP peptides bind to the C-terminal region of MYPT1, CCLR (residues 924–1030). Concentration-normalized time derivative g(s*) graphs of the MRIP peptides by themselves and in equimolar mixtures: Lines, CCLR (30 μM); +, individual MRIP peptides by themselves (MRIP^545-878^, 14 μM; MRIP^724-878^, 50 μM; MRIP^724-837^, 67 μM); black filled-in circles, original equimolar mixtures (MRIP^545-878^, 14 μM each; MRIP^724-878^, 30 μM each; MRIP^724-837^, 67 μM each); open circles, 3-fold dilutions; gray filled-in circles, 9-fold dilutions.

AUC was performed to examine the interactions of MRIP^545-878^, MRIP^724-878^, or MRIP^724-837^ individually with the MYPT1 peptide (residues 924–1030) containing a putative coiled coil domain (CC) and the leucine repeat region (LR), designated as CCLR. All three MRIP/MYPT1 mixtures exhibited shifts in the concentration-normalized time derivative g(s*) curves to the right, indicating binding (Fig 3). In addition, the shifts in the curves were concentration-dependent, suggesting reversible interactions. Binding between MRIP^724-837^ and CCLR indicates that residues spanning CC2 and CC3 of MRIP are sufficient for the interaction.

The concentration ranges of the MRIP peptides in the mixtures overlap with those of the MRIP peptides by themselves (Fig 2). Thus, the greater s* values of the MRIP/MYPT1 mixtures than those of the individual MRIP peptides indicate that MRIP^545-878^ monomer, MRIP^724-878^ dimer, and the mixture of monomer and dimer of MRIP^724-837^ bind to CCLR. This in turn suggests that MRIP binds to MYPT1 either as a monomer or as a dimer. To obtain the binding stoichiometry, AUC was performed using MRIP^724-878^/CCLR mixtures at different ratios of the two peptides. MRIP^724-878^ was chosen because the AUC study yielded a good fit to a single species. In short, the binding stoichiometry was unattainable due to reversible self-association of CCLR [14], which made the system too complex to analyze.

### LR Containing the Residues 991–1030 of MYPT1 Is Sufficient for MYPT1 to Interact with MRIP

**Fig 4:**
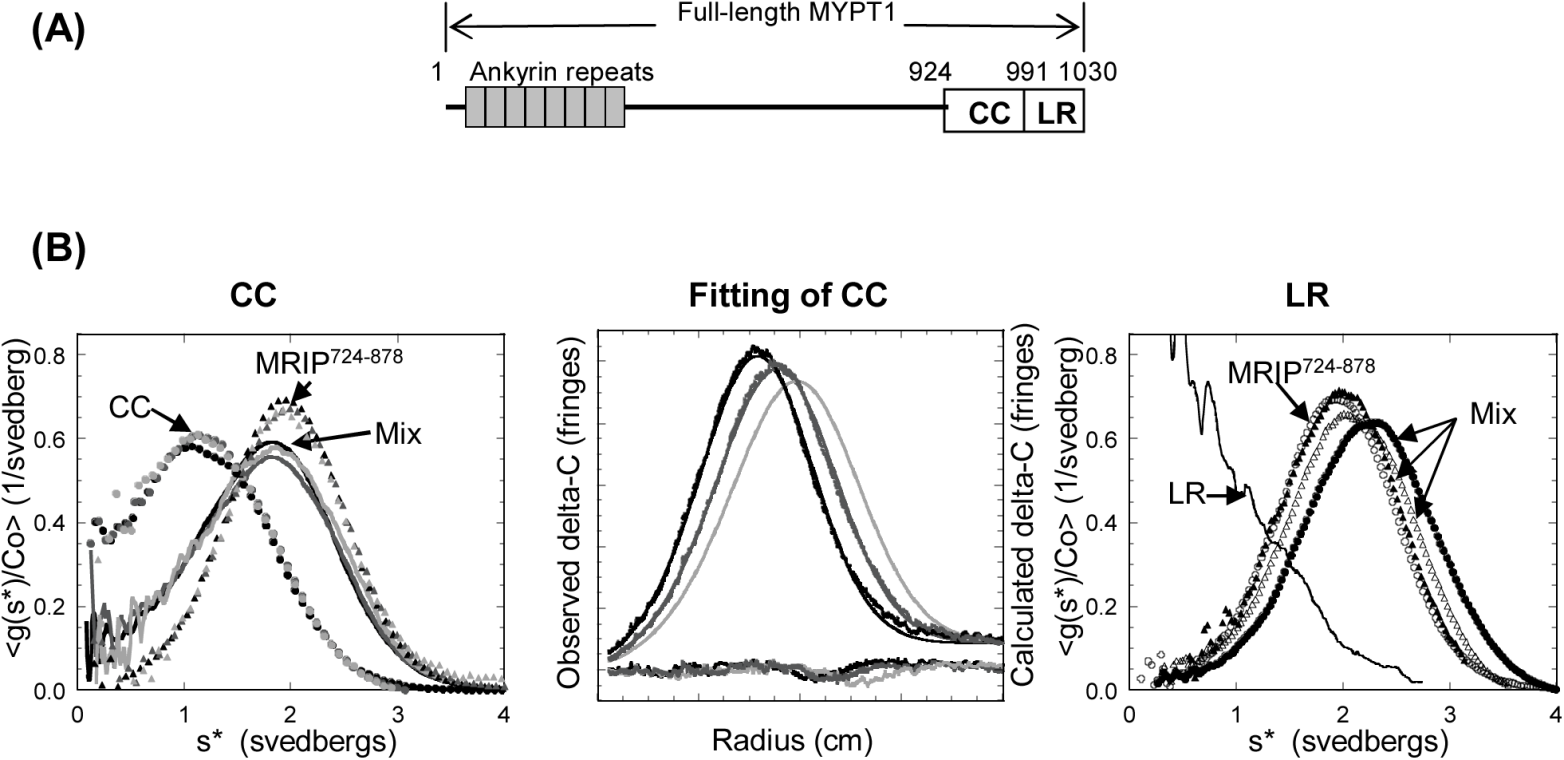
LR contains sufficient residues for the MYPT1/MRIP interaction. **(A)** Schematic diagram of subdomains of CCLR **(B)** CC does not bind to MRIP^724-878^ but LR does. **CC**; Concentration-normalized time derivative g(s*) plots of CC, MRIP^724-878^, and equimolar mixtures of the two peptides: Black filled-in circles, an original CC alone (107 μM); dark gray filled-in circles, a 3-fold dilution of CC alone; light gray filled-in circles, a 9-fold dilution of CC alone; black filled-in triangles, an original MRIP^724-878^ alone (50 μM); dark gray filled-in triangles, a 3-fold dilution of MRIP^724-878^ alone; light gray filled-in triangles, a 9-fold dilution of MRIP^724-878^ alone; a black line, an original equimolar mixture of MRIP^724-878^/CC (50 μM each): a dark gray line, a 3-fold dilution of the mixture; a light gray line, a 9-fold dilution of the mixture.

To determine the minimal MRIP-binding region of MYPT1, AUC was employed to examine interactions between sub-domains of CCLR, CC (residues 924–990) and LR (residues 991–1030) with MRIP^724-878^ (Fig 4A). CC is the putative coiled coil domain and LR contains 16 upstream amino acid residues (residues 991–1006) in addition to the leucine repeat (residues 1007–1028). MRIP^724-878^ was chosen because of its good fit to a single component, single-species model. The dilutions of CC alone, MRIP^724-878^ alone, or the CC/MRIP^724-878^ mixture were superimposable, indicating concentration-independence (Fig 4B; CC). An equimolar mixture of CC and MRIP^724-878^ showed no binding, the resulting curves being the simple sum of g(s*) curves for the individual components. Besides, g(s*) curves of dilutions were superimposable, suggesting no interaction. Global fitting of the mixture to a two-component model produced a good fit and the RMSD of the fit was 0.010 (Fig 4B; Fitting of CC). This suggests that there is no complex formation between CC and MRIP^724-878^. In contrast, an equimolar mixture of LR and MRIP^724-878^ showed a greater s* value than that of MRIP^724-878^ alone, indicating binding between the two (Fig 4B; LR). Moreover, the shift in the curve was concentration-dependent, suggesting a reversible interaction. A similar change in the g(s*) curve was observed from the mixture of MRIP^724-837^ and LR, confirming that LR contains sufficient residues for binding (data not shown).

To obtain the binding stoichiometry, a series of sedimentation equilibrium experiments was performed. MRIP^724-878^ was chosen due to its being a single species. Owing to the absence of aromatic residues, LR was employed. AUC was conducted using MRIP^724-878^ alone, LR alone, the 1:1 mixture of MRIP^724-878^/LR, and the 1:3 mixture of MRIP^724-878^/LR. Sedimentation data were collected both at 280 nm to detect only MRIP^724-878^ and at 220 nm to detect both. In the end, reversible self-association of LR [14] made the system too complex to obtain an unequivocal binding stoichiometry.

**Fig 5:**
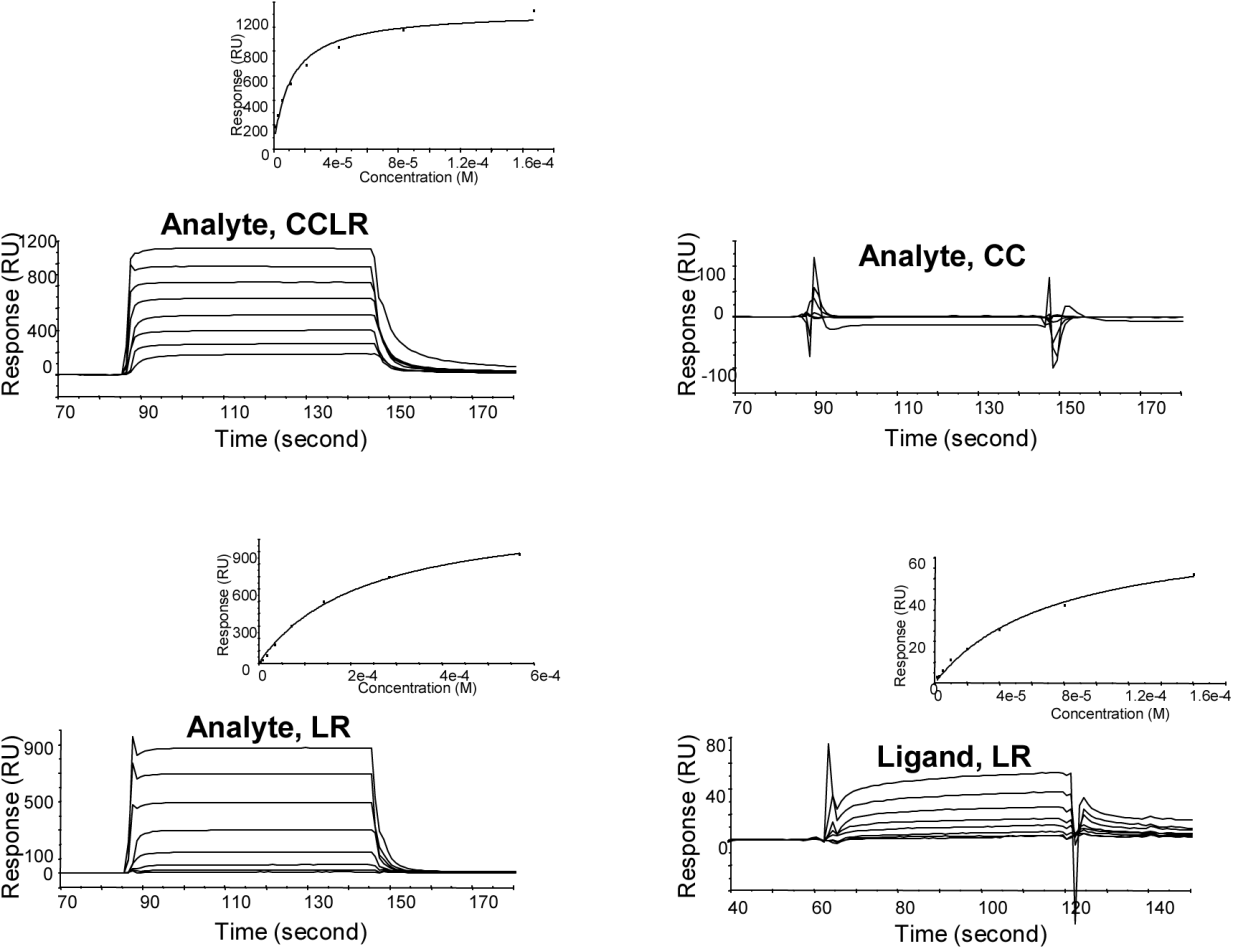
SPR analysis shows that LR has sufficient residues for the MYPT1/MRIP interaction. **Analytes CCLR, CC, LR**: Interactions between analytes, CCLR (167–1 μM), CC (318–10 μM), and LR (569–5 μM) with immobilized ligand, MRIP^724-878^ (1800 RU) after correction for nonspecific binding using G-actin (2230 RU). Shown is a representative out of 9 experiments for CCLR and 8 for LR in each, duplicates or triplicates. Insets show fittings of steady-state affinity. **Ligand LR**: Interaction of analyte, MRIP^724-878^ (85.6–0.7 μM) with immobilized ligand, LR (244.8 RU) after correction for nonspecific binding using a nebulin-derived peptide (600 RU). Shown is a representative out of 6 experiments.

To corroborate the results from AUC studies, SPR was employed. In accord with AUC analyses, CCLR and LR formed complexes with immobilized MRIP^724-878^ but CC did not (Fig 5; Analytes CCLR, CC, and LR). Equilibrium *K*_d_ values were obtained by nonlinear curve fitting of reference-corrected curves using the steady-state affinity fitting model in BIAevaluation version 4–1. The K_d_ of CCLR (encompassing CC and LR) was 14±3 μM and the K_d_ of LR was 195±31 μM. Thus, CCLR had higher affinity to the immobilized MRIP^724-878^ than LR even though CC did not interact with MRIP^724-878^. This indicates that upstream residues of LR may enhance the binding affinity although they cannot bind on their own.

The binding of MRIP^724-878^ to LR was demonstrated again in SPR experiments where a range of concentrations of MRIP^724-878^ was injected onto the immobilized LR (Fig 5; Ligand LR). The K_d_ was 40±19 μM for the dimeric MRIP^724-878^. It is of note that solution-based, free MRIP^724-878^ showed higher binding affinity than the immobilized one (40±19 vs 195±31 μM). This could be explained by the fact that free, solution-based MRIP^724-878^ might have preserved homodimers better than the amine coupled. Consequently, we speculated that the MRIP^724-878^ dimer might have higher MYPT1-binding affinity than its monomer.

### Glutamic Acids within LR Provide Additional Binding Affinity

**Fig 6:**
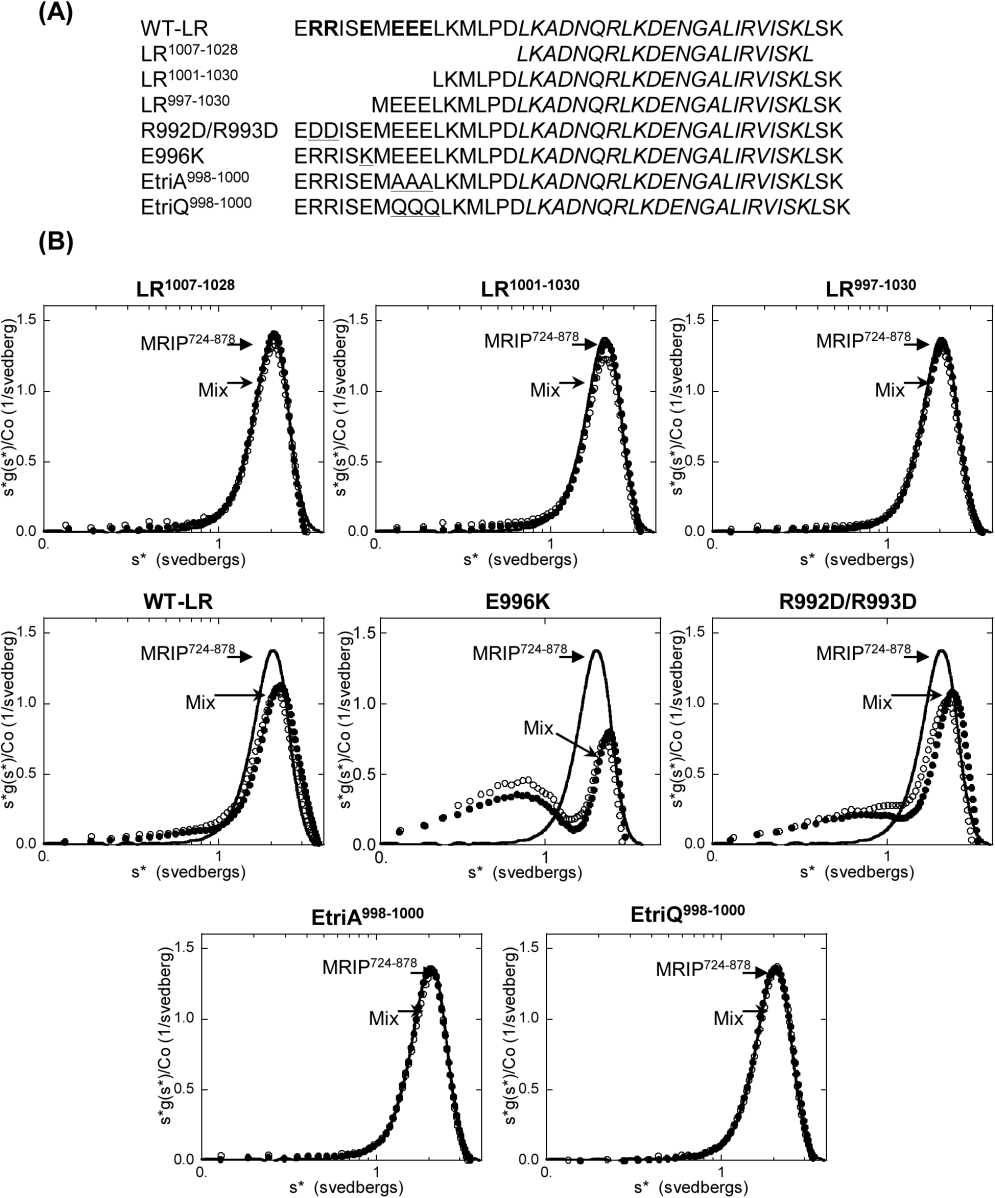
Glutamic acids within LR enhance the MRIP-binding affinity. **(A)** Amino acid sequences of the wild-type LR (WT-LR) and mutant LR peptides. Charged residues are in bold, the leucine repeat is in Italic, and substitution mutations are underlined. **(B)** Time derivative s*lg(s*) graphs: Line, MRIP^724-878^ alone (50 μM); filled-in circles, original mixture of MRIP^724-878^/each mutant LR peptide (50 μM each except for R992D/R993D and E996K whose mixtures consisted of excess amount of the mutant LR peptides); open circles, a three-fold dilution of the original mixture. The molar mass of the complex differs slightly from that of MRIP^724-878^ dimer (37.9 kDa vs 37.4 kDa), and thus the shift in the main peak position is not easily discernable in many cases.

The coiled coil is a common protein-protein interaction domain. The MRIP peptides in this study have putative coiled coil domains and are α-helical. In contrast, the MYPT1 peptide, LR is not α-helical even though it contains the leucine repeat [14]. Thus, we hypothesized that charged residues of MYPT1 might participate in the MYPT1/MRIP interaction with its leucine residues. We generated various LR peptides representing mutations in charged residues (Fig 6A) and examined their bindings to MRIP^724-878^ (Fig 6B). AUC analyses showed that the binding affinity of mutant LR peptides that were shorter than LR in length (LR^1007-1028^, LR^1001-1030^, and LR^997-1030^) decreased. Among mutant LR peptides with the same length as the wild-type but with substitutions for charged residues, only those mutants in which glutamic acid residues 998–1000 were replaced by alanine (EtriA) or glutamine (EtriQ) showed significantly decreased binding affinity. That is, the main peak positions of the mixture of R992D/R993D and E996K were shifted to the right, indicating binding. In contrast, the mixtures of EtriA and EtriQ showed g(s*) curves similar to that of MRIP^724-878^ alone, indicating weaker binding. This in turn suggests that the negative charges of glutamic acids enhance the binding affinity toward MRIP^724-878^ since glutamine differs from glutamic acid only in its absence of charge.

SPR was performed to corroborate AUC analysis. SPR binding profiles between MRIP^724-878^ and various LR mutants agreed with the AUC results (data not shown). The relative affinities were specific but weak enough to be at the limit of detection on the instrument. This precluded a model of a steady-state affinity and thus the K_d_ values were unattainable.

## Discussion

Using AUC, we demonstrated that MRIP can form a homodimer and binds to MYPT1 as a monomer or as a dimer. It is known that scaffold proteins organize protein complexes to facilitate interaction between signaling components. It could be envisioned that the monomeric MRIP complex would represent one status and the dimeric MRIP complex another. Perhaps the MRIP dimer may serve as a scaffold protein better than its monomer by providing better stability, specificity, complexity, and simultaneous accommodation of multiple proteins. In fact, our SPR data showed that the steady-state affinity for solution-based MRIP^724-878^ was higher than that of the immobilized (40±19 vs 195±31 μM), indicating that dimerization of MRIP might increase the MYPT1-binding affinity. However, more work will be required to confirm roles for the MYPT1/monomeric MRIP and the MYPT1/dimeric MRIP complexes in the cell. The buffer employed in this study is close to the pH and salt concentrations of physiological buffers, and thus the results should be relevant to physiological conditions.

Our study indicates that the shortest MRIP peptide, MRIP^724-837^ (CC2-CC3) possesses sufficient residues for dimerization, being consistent with the results of others’ immunoprecipitation/immunoblotting studies [7,12]. Moreover, the study showed that MRIP^724-878^ (CC2-CC4) was a tight dimer, MRIP^724-837^ (CC2-CC3) a mixture of monomer-dimer, and MRIP^545-878^ (CC1-CC4) a monomer. This can be interpreted that the dimerization affinity is MRIP^724-878^ (CC2-CC4) > MRIP^724-837^ (CC2-CC3) > MRIP^545-878^ (CC1-CC4), indicating that CC2 and CC3 comprise a fundamental dimerization domain, and CC4 facilitates dimerization. The presence of CC1 seems to obstruct dimerization. It is of note that MRIP mutations employed by Mulder *et al*, L905P/I912P and I919P/L926P showed little oligomerization [12]. According to our coiled coil analysis, these mutations were double point mutations in CC5. The lack of oligomerization of these mutations is not surprising since incorporation of two proline residues into CC5 is likely to result in severe constraints in the CC5 backbone perhaps disrupting the coiled coil structure. This leads us to speculate that CC5 and CC6, while not necessary for dimer formation per se, may nevertheless enhance dimerization. Thus, the full-length MRIP (CC1-CC6) may be able to form a dimer. Originally, we chose not to express the full-length MRIP because our study focused on interaction sites between MRIP and MYPT1. However, we decided to test directly our hypothesis on the potential roles of CC5 and CC6 using a purified MRIP peptide spanning resides 545–929 (CC1-CC5) and the full-length MRIP protein (CC1-CC6). We expressed and purified the MRIP peptide (residues 545–929) and the full-length MRIP protein using a chitin-binding tag and a GST-tag, respectively in bacteria. However, the expression of the MRIP peptide (residues 545–929) was so low that 10 L of bacterial culture did not yield enough peptide for an AUC experiment. The GST-tagged full-length MRIP was expressed in bacteria but the protein precipitated after affinity purification. This indicates that the MRIP peptide (residues 545–929) and the full-length MRIP protein require a eukaryotic expression system to obtain adequate material for study, which we hope to explore in the future.

Our AUC study showed that MRIP^545-878^, MRIP^724-878^, and MRIP^724-837^ bound to MYPT1, suggesting that the residues 724–837 of MRIP (represented by MRIP^724-837^) are sufficient for the MYPT1/MRIP interaction. It also showed that LR containing the residues 991–1030 of MYPT1 contains sufficient residues for the MYPY1/MRIP interaction. Thus, our results agree with those of others’ immunoprecipitation/immunoblotting [7,11]. Our results, however, differ from those of Mulder *et al* in which L857, I912, and I919 were critical for MRIP to interact with MYPT1 [12]. Our MRIP peptides lacking the aforementioned residues bound to MYPT1. This in turn suggests that none of the three residues are critical for complex formation.

We demonstrated that the Glu residues of MYPT1 participate in the MYPT1/MRIP interaction with its leucine residues. Our data showed that the C-terminal region of MYPT1, LR bound to MRIP^724-878^ and the Glu substitutions of LR decreased the binding affinity. This indicates that the Glu residues, if not directly involved in binding, may stabilize the peptide structure and that the charge differences derived from the Glu substitutions can produce some kind of allosteric effect that modifies the tertiary structure in the region of LR. Consequently, the Glu residues provide extra MRIP-binding affinity to LR at which MYPT1 interacts with other proteins also. We hope to confirm the different MRIP-binding affinities between the wild-type LR and the Glu substitutions of LR using their full-length MYPT1 versions in the future. For measuring the interaction, SPR and AUC are preferable to immunoprecipitation due to limitation in sensitivity. The leucine residues within LR are important for the MYPT1/MRIP interaction. Therefore, we speculate that the Glu substitutions of the full-length MYPT1 may interact weakly with MRIP via the leucine residues and thus will be co-immunoprecipitated with MRIP. The full-length MYPT1 requires a eukaryotic expression system. Protein kinase G is known to interact with the same leucine residues within LR. It remains to be determined whether the Glu residues participate in the interaction with protein kinase G.

In short, this study explains the biophysical properties of the MYPT1/MRIP interaction and establishes that the Glu residues of MYPT1 enhance the MYPT1/MRIP interaction.

## Materials and Methods

### Preparation of Peptides

The cDNA fragment encoding MRIP peptides were amplified using a full-length MRIP cDNA as a template with the following primer pairs: MRIP^545-878^, 5’-GGGCATATGGCTGAGTTCCGTCCCATC/5’GGGGAATTCTTACAGCGTCCGCAACCGTG; MRIP^724-878^, 5’-ACGTGCCATATGGAGCGAGGGTTTGCAGCAATG/5’-GGGGAATTCTTACAGCGTCCGCAACCGTGT; MRIP^724-837^, 5’-ACGTGCCATATGGAGCGAGGGTTTGCAGCAATG/5’-GGGGAATTCTTAGGCCAGATGGGCATTCTC. The PCR products were ligated into pCR2.1-TOPO using the TA cloning system and sequenced in both directions. The IMPACT™ (Intein Mediated Purification with an Affinity Chitin-binding Tag) Kit (New England BioLabs) was used to express peptides in bacteria to obtain tag-free peptides. The PCR amplicon encoding MRIP peptides were fused in frame to the chitin binding domain of pTYB12 vector utilizing the NdeI/EcoRI restriction sites. The expression vectors for the MYPT1 peptides, CCLR (residues 924–1030), CC (residues 924–990), and LR (residues 991–1030) were constructed as described [14]: In the reference literature, CCLR and LR were named CCLZ and LZ, respectively. Recombinant peptides were produced in *Escherichia coli* strain BL21 (DE3) and purified using chitin beads according to the manufacturer's protocol. For further purification, the eluent from chitin beads was loaded on a Vydac C8 reversed phase column and subjected to HPLC using an acetonitrile gradient. Purity and identity of the peptides were confirmed by polyacrylamide gel electrophoresis (PAGE) and mass spectrometry. For the mutagenesis study of LR, the wild-type and mutant LR peptides were synthesized, purified by reverse-phase HPLC, and lyophilized to dryness at the peptide core in Boston Biomedical Research Institute.

### Analytical Ultracentrifugation (AUC)

Sedimentation velocity experiments were done on a Beckman Instruments Optima XL-I analytical ultracentrifuge equipped with Rayleigh optics. The cells were equipped with sapphire windows and 12 mm charcoal-filled Epon centerpieces. Apparent sedimentation coefficient distribution patterns were computed by the time derivative method [15–17]. Peptides were dialyzed against 50 mM potassium phosphate buffer (pH 7.4) containing 100 mM KCl, and 1 mM DTT at 4°C overnight and then subjected to AUC at 50,000 rpm and 20°C. Molar mass was computed from sedimentation velocity profiles using SedAnal [18], which uses a nonlinear least-squares curve fitting algorithm to fit data to solutions of the differential equation (the Lamm equation) describing sedimentation. Fits were performed on time difference data to remove the time-independent systematic baseline components [18]. Values of *s* and *D* produced by the fitting procedure were substituted into the Svedberg equation to obtain the molar mass of the protein, *M*:

$$M=\frac{RT}{\left(1-\bar{\nu }\rho \right)}\frac{s_{20,w}^{o}}{D_{20,w}^{o}}$$

where *ρ* is the density of the buffer and $\bar{\nu }$ is the partial specific volume of the protein. Sednterp [19] was employed to compute values of *ρ* and $\bar{\nu }$ from the amino acid sequence.

### Surface Plasmon Resonance (SPR)

Binding analyses of SPR were conducted on a Biacore 3000 (GE Healthcare) at 25°C. Sensor chips and reagents for immobilization (1-ethyl-3-(3-dimethylaminopropyl) carbodiimide, N-hydroxyl-succinimide, and ethanolamine) were purchased from GE Healthcare. Peptides were immobilized onto a CM5 sensor chip at 232–5841 resonance units (RU) using amine coupling. Binding studies were done using two-fold serial dilutions of the peptides in 50 mM potassium phosphate buffer (pH 7.4) containing 100 mM KCl. Actin, CC, or a nebulin-derived peptide was immobilized at similar densities to serve as a negative control surface. BIAevaluation software version 4–1 (GE Healthcare) was used for data analysis. Equilibrium *K*_d_ values were obtained by nonlinear curve fitting of reference-corrected curves using the steady-state affinity fitting model in BIAevaluation version 4–1. *K*_d_ values are reported as mean and standard deviation.

## Abbreviations

AUC: analytical ultracentrifugation
CC: coiled coil; Glu, glutamic acid
LR: leucine repeat region
MRIP: myosin phosphatase-RhoA interacting protein
MYPT1: myosin phosphatase target subunit
SPR: surface plasmon resonance
